# Implementation of wedged-serial protein crystallography at PROXIMA-1

**DOI:** 10.1107/S1600577521013242

**Published:** 2022-01-17

**Authors:** Igor Chaussavoine, Tatiana Isabet, Robin Lener, Pierre Montaville, Ramakrishna Vasireddi, Leonard M. G. Chavas

**Affiliations:** a Synchrotron SOLEIL, 91192 Gif-sur-Yvette, France; b Nagoya University, Nagoya 464-8603, Japan

**Keywords:** serial crystallography, microfluidic, PROXIMA-1

## Abstract

An approach for serial crystallography experiments based on wedged-data collection is described. This is an alternative method for recording *in situ* X-ray diffraction data on crystalline samples efficiently loaded in an X-ray compatible microfluidic chip. Proper handling of the microfluidic chip places crystalline samples at geometrically known positions with respect to the focused X-ray interaction area for serial data collection of small wedges. The integration of this strategy takes advantage of the greatly modular sample environment available on the endstation, which allows access to both *in situ* and more classical cryo-crystallography with minimum time loss. The method represents another optional data collection approach that adds up to the already large set of methods made available to users. Coupled with the advances in processing serial crystallography data, the wedged-data collection strategy proves highly efficient in minimizing the amount of required sample crystals for recording a complete dataset. From the advances in microfluidic technology presented here, high-throughput room-temperature crystallography experiments may become routine and should be easily extended to industrial use.

## Abstract 2

The implementation of microfluidic-based wedged-serial protein crystallography at PROXIMA-1 opens new possibilities for *in situ* data collection experiments with low sample consumption.

## 1. Introduction

Serial protein crystallography at synchrotrons has grown in interest following the developments at X-ray free-electron laser (XFEL) sources for X-ray diffraction data collection (Grünbein & Kovacs, 2019 ▸; Chavas *et al.*, 2015 ▸) with reduced damages (Nass, 2019 ▸), a serious obstacle for sensitive biological samples. For the XFEL approach, in contrast to the more classical synchrotron cryo-crystallography, hundreds of thousands of *in situ* crystals are exposed to extremely intense X-rays that lead to a unique diffraction image recorded per crystal using the principle referred to as ‘diffraction before destruction’ (Neutze *et al.*, 2000 ▸; Johansson *et al.*, 2017 ▸). The great need for, and consumption of, samples represent the main caveat of this XFEL approach, classically requiring between 100 000 and several million crystal diffraction images to confidently solve a macromolecular structure (Chapman *et al.*, 2011 ▸; Chapman & Fromme, 2017 ▸). XFEL and synchrotron sources differ in the nature of the photons being distributed, which, in turn, affects the type of studies that can be performed, the overall instrumentation on the experimental station, how the sample should be prepared and handled, and how data analysis needs to be carried out. Instrumental developments, however, are often shared or adapted, especially when referring to sample preparation and handling. By taking advantage of technological improvements related to the speed of data recording, size and quality of X-ray beams, identification and handling of smaller samples, and data processing methods, strong interest has grown at synchrotron facilities for employing a variant of XFEL serial crystallography as a more standardized method for protein crystal X-ray diffraction (Pearson & Mehrabi, 2020 ▸; Martin-Garcia, 2021 ▸; Diederichs & Wang, 2017 ▸; Owen *et al.*, 2017 ▸).

A variety of strategies to study crystals at synchrotron and room temperature has led to the engineering of approaches that can be divided into moving-target and fixed-target systems (a non-exhaustive list of strategies is reported Table 1 ▸). The concept of imaging X-ray damage-free structures while applying intense beams at third-generation synchrotrons was first explored using capillaries and in-flow sample delivery (Stellato *et al.*, 2014 ▸). Rather than capillaries, microfluidic chips were also interfaced for diffraction measurements using a combination of continuous crystal flow and small-wedged rotation (Monteiro *et al.*, 2020 ▸). The implementation of high-viscosity extrusion injectors represents yet another example of moving targets (Botha *et al.*, 2015 ▸), with a real advantage of the technique when considering time-resolved experiments. The above examples efficiently replenish samples, and generally do not require external instrumentation; however, the position and orientation of the samples remain unknown which renders the data processing somewhat complicated.

Taking advantage of automation and state-of-the-art goniometry implemented at macromolecular crystallography beamlines, great efforts are provided in developing data collection strategies from fixed-target systems. The lead innovation flag behind these sample-delivery techniques targets a reduction in sample consumption. The conveyor belts approach (Soares *et al.*, 2014 ▸) returned data comparable to mesh-loops with a great potential for reducing the solvent around the crystals for an improved signal-to-noise ratio, although aggregation of crystals was reported and may affect data quality. The use of patterned chips represents most of the recent engineering observed by various groups. Among the various methods already implemented at synchrotrons, Roedig *et al.* (2016 ▸, 2017 ▸) presented a silicon-based handling chip optimized for high-speed data collection of crystal samples presented on a fixed target, at both synchrotron and XFEL facilities. The preparation protocol of the chip and its nature induced a stochastic orientation and positioning of the crystals, which lead to hit rates at XFELs of 2% to 10%, yet representing a comfortable ratio for ‘placed crystal’ over ‘diffracted crystal’ higher when compared with other sample-delivery approaches.

In order to improve data quality and open the field to a larger set of samples, we have chosen to concentrate on yet another method for crystal delivery, based on handling crystal samples by microfluidic devices. In previous studies, Lyubimov *et al.* (2015 ▸) introduced a technique that reduces sample consumption by placing the crystals to be diffracted at known positions within a microfluidic chip. The technique drastically reduces crystal usage while preventing dehydration of samples and performing the experiments at room temperature. The device can be adapted to either XFEL or synchrotron experiments; however, specific hardware adaptations are required to operate the chips in the sample environment. One of the biggest advantages in experimenting with these devices lies in the possibility to perform multi-crystal diffraction data collection, which greatly reduces the impact of radiation damage to the sample (Gotthard *et al.*, 2019 ▸). Most surprisingly, the technique introduced by Lyubimov and coworkers has not been reported as extensively used, although it represents an optimized solution for fast and assisted positioning of the crystals at the synchrotron X-ray beam interaction point.

In line with the goal of minimizing sample loss, the microfluidic technology is, in essence, based on very low consumption of injected materials. When applied to macromolecular crystallography, microfluidic chips have to be compatible with the diffraction experiments, often framed by thin films composed of elements that weakly absorb X-rays. Depending on the design of the chip, the loading of samples may be delicate and eventually become a challenging process. Additionally, the highly random distribution of crystals within the microfluidic chips complicates data collection, classically performed without considering the individual positions of the crystals and exposing them to the X-ray beam in an unsystematic manner.

In the current work, a simplified design of the Lyubimov *et al.* (2015 ▸) approach was engineered to isolate crystalline samples at positions geometrically known within the microfluidic device. Using the chip as a sample holder for tens-to-hundreds of crystals, full datasets on test samples were recorded at room temperature with data collection performed over a small rotation angle for each crystal position, with low-dose exposition and continuous rotation for an easier indexing of the reflections. During each data collection, the microfluidic chip is not translated, and the crystals are fully centered at the X-ray interaction area while being rotated. Owing to the low X-ray absorption of the chip, the data collection method demonstrates efficient *in situ* diffraction collection with low sample consumption. Additionally, the small physical dimensions of the device minimize the risk of hardware collision with the beamline equipment while recording larger rotation angles. Taken together with its applicability to diffraction experiments for macromolecular crystallography, this opens the possibility of implementing the chip at most synchrotron MX beamlines equipped with a goniometer head accepting magnet-type sample holders.

## 2. Material and methods

### 2.1. Sample preparation

Lysozyme crystals were prepared in batches to generate a range of sizes with optimized dimensions of 15 µm for the longest direction. Lysozyme powder (Sigma–Aldrich) was dissolved in a buffer of 50 m*M* acetate mixed with the mother liquor solution (1 *M* NaCl, 35% ethyl­ene glycol, 12.5% PEG3350, 50 m*M* acetate), incubated for 15 h at 283 K before storage at 253 K.

Insulin crystals were produced from a powder (Sigma–Aldrich) dissolved in 10 m*M* HCl, 50 m*M* citrate and 6 m*M* zinc sulfate and pre-incubated at 323 K for 20 min. The solution was then supplemented by 15% acetone, incubated for an additional 20 min at 323 K and the crystallization was quenched by an incubation at 293 K for 6 h before storage at 277 K.

### 2.2. Microfluidic chip manufacturing

**Figure 1:**
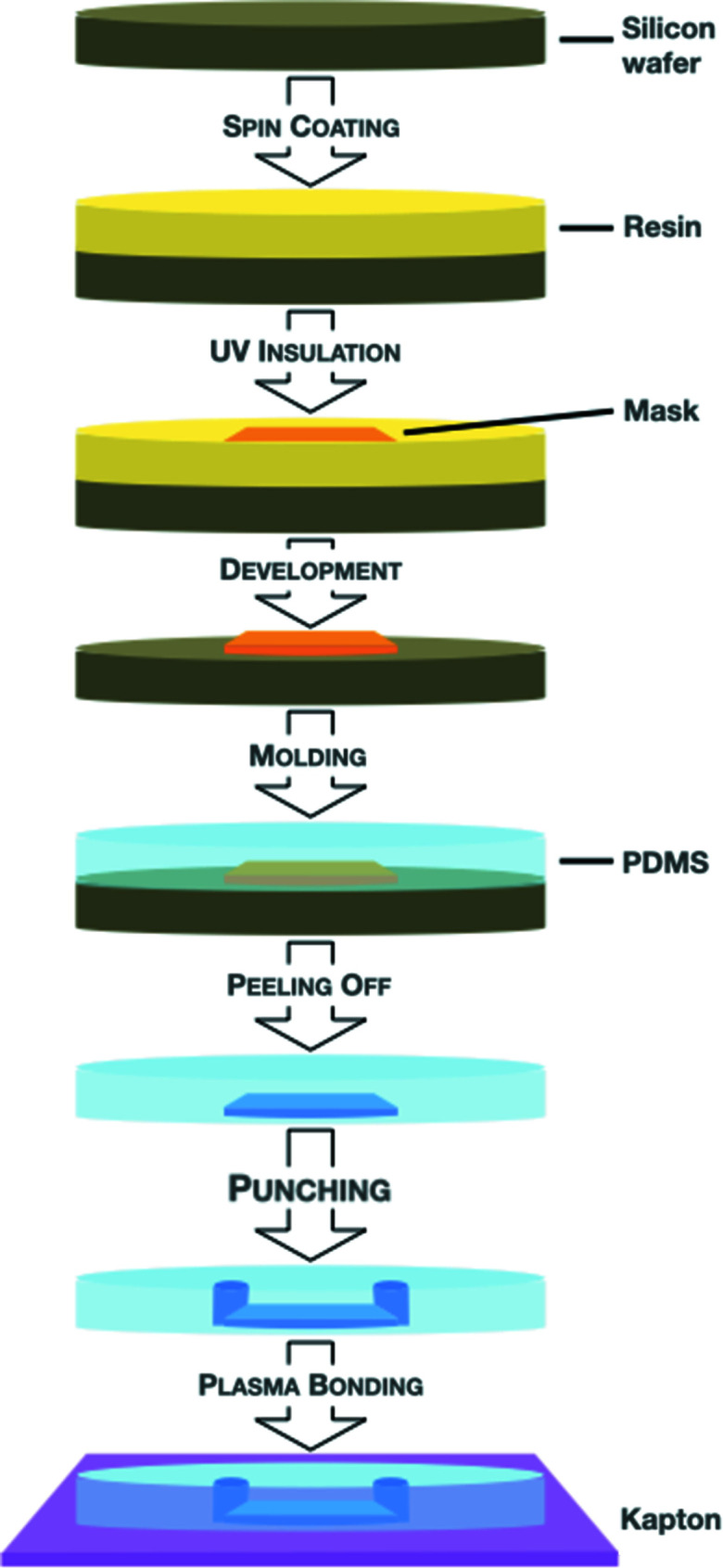
Scheme of the various steps involved in manufacturing the chip.

The chip was manufactured in a stepwise process using standard methods of spin-coating and plasma bonding (Fig. 1 ▸). The trapping pattern was UV-insulated on the 40 µm-thick spin-coated resin (SU-8 2015) using a laser writer (KLOE). After development of the resin and PDMS molding, holes for the inlets and outlets were punched and a final step of plasma bonding on a 25 µm Kapton support hermetically closed the chip. The design of the chip was optimized to trap crystals measuring 15 µm to 50 µm (longest direction). The trap channel width is 10 µm, leading to difficulty in trapping samples smaller than 15 µm. With the current design, about 20% of the manufactured chips were deficient while presenting leaks when injecting buffers, most likely caused by operational fluctuations of the equipment employed to make the chips.

### 2.3. Sample injection

Great care was taken while injecting the samples inside the chip, to avoid unnecessary mechanical stress on the crystals and favor efficient trapping. The most reproducible injection protocol results from the use of a pressure controller (Fluigent) for the injection of the crystal-containing solution into plastic tubing of 0.5 mm inner diameter. For an efficient loading, the sample-free crystal solution was first injected with a pressure of 50 mBar for long periods (10 h) to remove all potential air bubbles within the channels (see Fig. S1 and Movie S1 of the supporting information). When ready, the chip was then loaded with the samples after gentle mixing to minimize sedimentation of the crystals. Before mounting on the beamline goniometer head, the chip was inspected for the presence of crystals in the traps under microscopes.

### 2.4. Chip handling

Handling of the microfluidic chip was performed at the PROXIMA-1 beamline of Synchrotron SOLEIL, France (Chavas *et al.*, 2021 ▸). The final PDMS chip loaded with crystals is 20 mm in width and 30 mm in height. It is accommodated inside an adapted 3D-printed frame for further mounting on a magnet-type goniometer head (Fig. S2). Though with a different design, use of a 3D-printed frame has already been reported (Broecker *et al.*, 2018 ▸; Huang *et al.*, 2020 ▸) and presents the advantage to rigidify the chip, which allows easier handling and eventually robot-assisted mounting. Additionally, and most importantly, using such a tool reduces the physical stress and, in turn, avoids liquid/sample movements inside the chip during manipulation. Based on the geometry of the frame, the sample environment at the beamline was adapted to avoid any potential collision of hardware with the rotating chip.

### 2.5. *In situ* geometrically optimized raster scanning

The microfluidic chips were designed to place the samples at known spatial positions to optimize *in situ* data collection at each position without spending time to re-center. *In situ* geometrically optimized raster (IGOR) scanning was implemented within an optional package of the *MXCuBE* GUI (Gabadinho *et al.*, 2010 ▸) specifically designed for this purpose (code available on request). In brief, the chip mounted on the goniometer head is pre-centered on three fiducials, usually represented by crystal positions marked and understood within *MXCuBE* as being the alignment points for the chip. Based on the recorded positions of these three marks, *MXCuBE* can calculate the coordinates for all the traps within the chip and uses these coordinates to automatically and sequentially position all the crystals at the X-ray interaction area. Wedges of a few degrees are then recorded at each position.

### 2.6. Data processing, analysis and structure determination

All data collections were performed at PROXIMA-1 (Chavas *et al.*, 2021 ▸). Data on lysozyme crystals were collected at room temperature (∼294 K) on a single row of 30 crystals by recording wedges of 30° per crystal, with oscillations of 0.1° and an exposure time of 0.10 s on a Pilatus-6M (Dectris, Ltd). To avoid strong damages to the *in situ* crystals, the X-ray full beam at 12.67 keV energy (∼2 × 10^11^ photons s^−1^) was attenuated to ∼5 × 10^9^ photons s^−1^, which corresponds to a dose of approximately 58 kGy per crystal, below the recommended dose for *in situ* data collection (de la Mora *et al.*, 2020 ▸). Data on insulin crystals were collected using the IGOR scanning procedure by recording wedges of 10° with 0.05° oscillations at a frequency of 20 Hz, on an EigerX-16M (Dectris) at 12.67 keV energy and for an attenuated X-ray beam of ∼5 × 10^9^ photons s^−1^.

Data were processed with *XDS* (Kabsch, 2010 ▸) through the *autoProc* package (Vonrhein *et al.*, 2011 ▸). Datasets were converted to MTZ format by *POINTLESS* (Evans, 2005 ▸), and scaled and merged by *AIMLESS* (Evans & Murshudov, 2013 ▸), as implemented within the *autoProc* procedure, taking *CC*_1/2_ ≥ 0.85 and *I*/sig(*I*) ≥ 2.5 as the resolution cutoff criteria in the outermost resolution shell. Calculations for dose deposition were performed using *RADDOSE-3D* (Zeldin *et al.*, 2013 ▸).

Structure-factor amplitudes were obtained by *TRUNCATE* (French & Wilson, 1978 ▸). Rotational and translational functions were calculated and compared by *MOLREP* (Vagin & Teplyakov, 2010 ▸) using the coordinates of the PDB entries 4dt3 (Cha *et al.*, 2012 ▸) and 3w7z (Hoshikawa *et al.*, unpublished) as template models for lysozyme and insulin, respectively. The solved structures were then run through rounds of refinement with *BUSTER* (Bricogne *et al.*, 2017 ▸), and manual model buildings using *Coot* (Emsley *et al.*, 2010 ▸). Data analysis and refinement statistics are shown in Table 2 ▸.

## 3. Results and discussion

**Figure 2:**
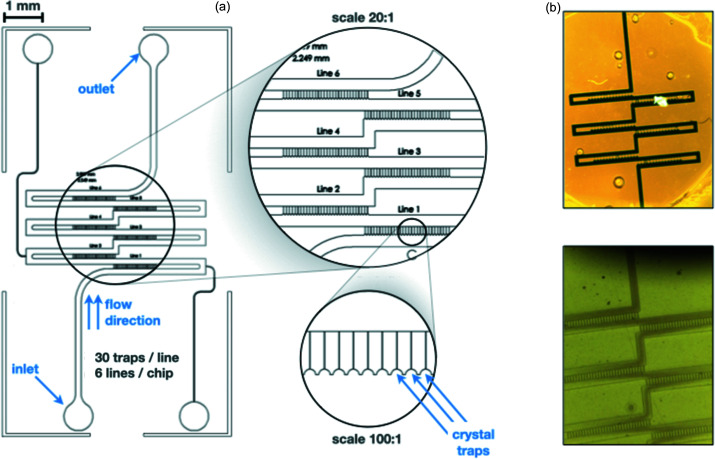
Design of the trapping chip. (*a*) CAD-drawing of the chip, highlighting the arrangement of 30 traps within each of the 6 lines contained in the device. Inlet, outlet, flow direction and trap locations are indicated in blue. (*b*) Images of two trapping chips loaded with different colorant to increase the contrast and highlight the design.

In the course of this work, the microfluidic chips developed for the IGOR scanning have been subject to constant improvement. Among the numerous versions of the device, efforts were made to both decrease the thickness of the chip for improving its response to X-ray exposition and optimize the geometry of the channels to decrease the risk of clogging while increasing the probability of trapping (Fig. 2 ▸). In an optimized version, the chip is made of 180 independent traps arranged in three double-rows; this block element can be multiplied to scale up the number of traps; however, the injection protocol may need to be adapted. Both the geometry of the channels and the thickness of the chip (100 µm in the beam direction) are optimized for crystals of dimensions ranging from 15 µm to 50 µm in the longest direction.

**Figure 3:**
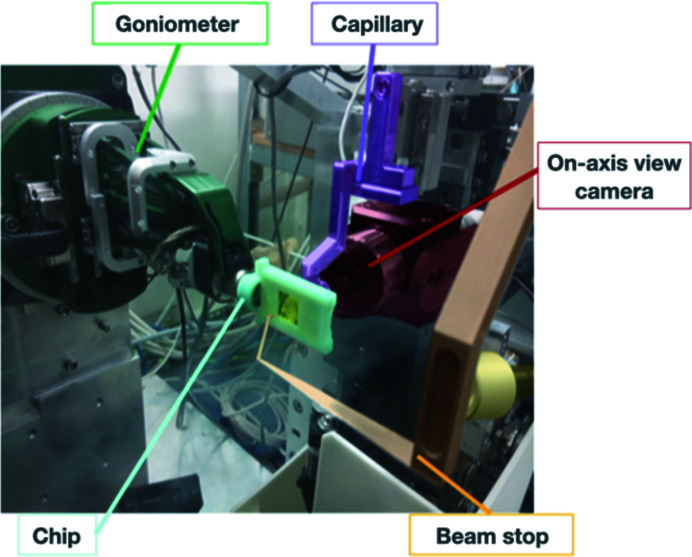
PROXIMA-1 beamline sample environment with a microfluidic chip mounted on the goniometer. The capillary and the beam stop are inserted with the cryostream retracted, the beamline is configured for data collection and the microfluidic chip is encapsulated within a 3D-printed frame to assist handling.

In order to acquire high-quality X-ray diffraction data from the trapped samples, the chip has to be centered to the X-ray interaction point with micrometer precision using the available goniometer. The reduced thickness of the chip makes it highly bendable and therefore increases the need for a stiff handling structure, which takes the form of an interface between the chip and the goniometer to ensure effective manipulation. Such a procedure was used and reported previously for other *in situ* applications (Broecker *et al.*, 2018 ▸; Huang *et al.*, 2020 ▸). In the current work, a specific 3D-printed encapsulating frame was designed to give a rigid support that protects the chip from mechanical stress and deformation and permits the chip to be gripped and lifted either manually or by a sample-mounting robot (Fig. S2). The addition of a magnetic cap allows accommodation on the conventional goniometer head (Fig. 3 ▸).

Centering and handling of both the microfluidic chip mounted on the goniometer and the crystals within is performed through the *MXCuBE* user interface. To facilitate centering of all the traps with the minimum actions, the original version of *MXCuBE* has been modified with options where the chips can be handled and IGOR scans can be scheduled (the details of these modifications will be described elsewhere). As a result of a 3-point centering operation, all the traps are recognized and marked as potential positions for further data collection. The users then have the possibility to either select all the positions or only those at which data collection should be preferentially performed.

**Figure 4:**
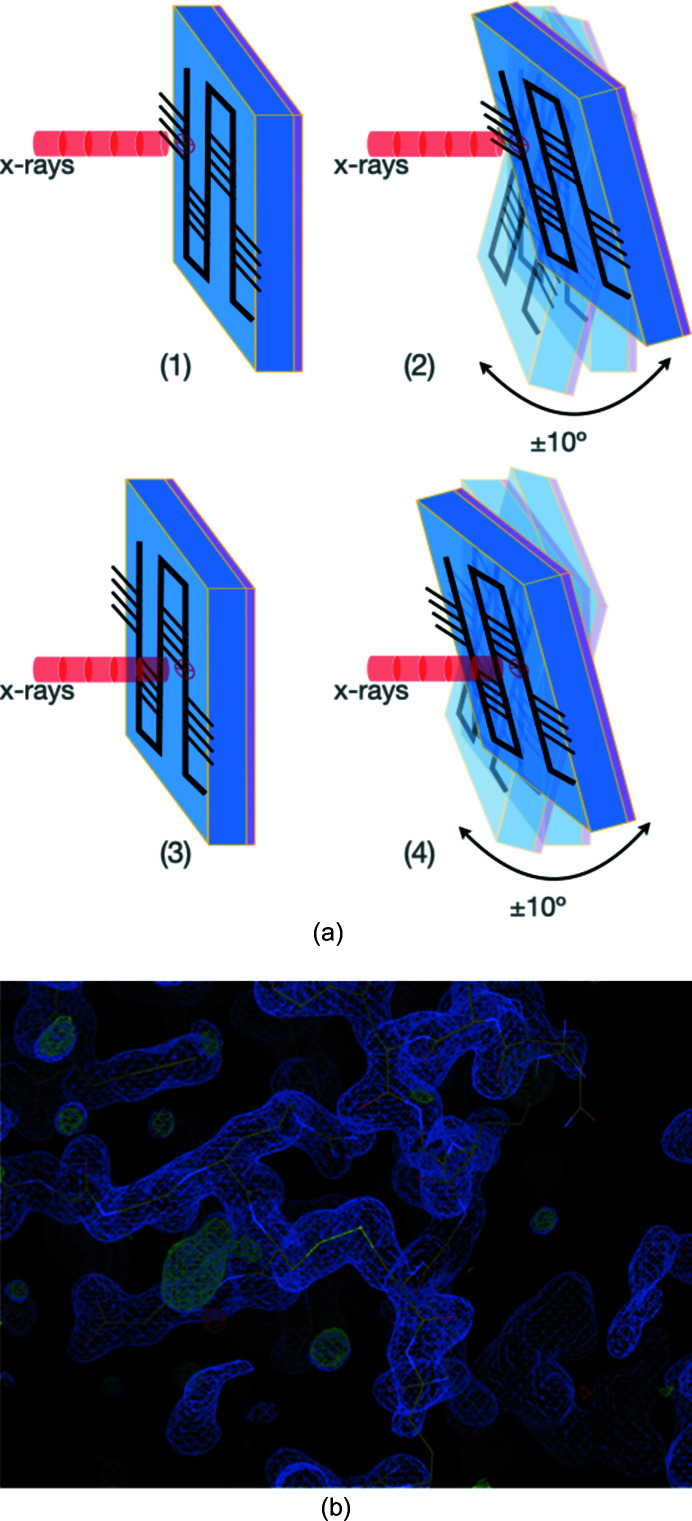
Data collection protocol and resulting electron density from the lysozyme crystal structure. (*a*) Data collection strategy using small wedges on microfluidic chips. (1) The first crystal position is brought to the beam, (2) followed by data collection of ±10° around the centered position. (3) The chip is then moved to another position and (4) data collection is repeated. (*b*) Electron density of the lysozyme structure obtained after structure determination and with no round of refinement.

The IGOR scanning method applies a simplified wedged-serial crystallography strategy of collecting X-ray diffraction data. Such a small-wedged data collection strategy was used early in the structural studies of sensitive membrane proteins (Cherezov *et al.*, 2007 ▸), and was recently successfully implemented within automated data collection pipelines at the ESRF and SPring-8 facilities (Zander *et al.*, 2015 ▸; Hirata *et al.*, 2019 ▸). In the current procedure, after moving the crystal to the center of the X-ray beam, fine-slice wedges of few degrees (±10° by default) are collected before moving to the next target [Fig. 4 ▸(*a*)]. Contrary to previously reported serial synchrotron rotation crystallography strategies (Gati *et al.*, 2014 ▸; Hasegawa *et al.*, 2017 ▸; Roedig *et al.*, 2017 ▸), the X-ray beam fast-shutter is closed during the centering of the crystals and the goniometer position is oriented back to its starting angle before each X-ray exposure. Data collection on all crystals within the chip takes consequently longer; however, the choice was made to minimize the number of photons delivered on the crystals to reduce unnecessary radiation damage while keeping the benefits of collecting continuous oscillation data.

Data processing of the serial crystallography data was performed manually, and data were selected for merging based on identical crystal lattice parameters. Overall, 9 out of 30 data were selected for lysozyme crystals, whereas many more data (a full chip or 180 traps) were collected for insulin for only 13 that could be merged. Phasing of lysozyme and insulin structures was performed with the sole purpose of confirming that no major issues occur within the protein structures; thus, a single run of restrained refinement followed the molecular replacement steps, with no additional rebuilding of sides chains nor inclusion of water molecules. The structures show no additional feature when compared with already well documented structures, with radiation damages clearly not visible from the electron density maps at these resolutions [Fig. 4 ▸(*b*)].

## 4. Conclusions

The exciting progress witnessed in the development of microfluidic chips applied to biological objects illustrates the great potential of these devices for handling samples that are classically difficult to manipulate, fragile or even hazardous. Operation of the microfluidic chips remains delicate, and great care should be provided when exposing the crystals to strong X-rays at room temperature. Additionally, the nature and choice of the compounds used to manufacture the chip will strongly affect the absorption of the incoming X-rays and resulting recorded noise, which will directly impair the quality of the recorded data. The current work introduces a low-absorption device; however, improvements should be provided to further minimize the background noise originating from the chip. The IGOR scanning approach appears as an effective solution to the merging of serial X-ray diffraction data, with indexing facilitated by an automated pre-centering of the crystals trapped in the chip and through recording continuous oscillation angles. This approach will remain of greater interest for samples with data classically difficult to index, weak to radiation damages or for which careful data collection protocols should be applied.

## Supplementary Material

Supplementary movie. DOI: 10.1107/S1600577521013242/yi5117sup1.mp4

Figures S1, S2 and S3. DOI: 10.1107/S1600577521013242/yi5117sup2.pdf

## Figures and Tables

**Table 1:** Major sample (protein crystals) delivery systems used at X-ray sources This comparative summary is inspired by Lyubimov *et al.* (2015 ▸) and represents various existing liquid-jet and fixed-target sample delivery methods notably used for serial femtosecond crystallography experiments at XFEL sources. The Efficiency column represents an estimation of the average number of crystals per integrated image based on the information provided in the reference.

Method	Efficiency	Advantages	Limitations
GDVN jet	25000 (Sierra *et al.*, 2012 ▸)	Low background, crystals in crystallization buffer	High sample consumption, possible clogging of the system, possible sample damage
Electrospinning jet	500 (Sierra *et al.*, 2012 ▸)	Low background, low flow rate	Require viscous media, potential impact of electrostatic charge on samples
LCP jet	500 (Weierstall *et al.*, 2014 ▸)	Low background, low flow rate, lipidic cubic phase	Crystals must grow in lipidic cubic phase
Micromesh	Not determined	Rapid data collection, small sample size, fits standard goniometer	Freezing and cryopreservation required, need multiple devices for complete datasets
Levitation droplets	2 (Roessler *et al.*, 2016 ▸)	Precise sample delivery, crystals in crystallization buffer	Solvent background scattering, evaporation
Silicon chips	11 (Roedig *et al.*, 2017 ▸)	Precise sample delivery, crystals in crystallization buffer	Evaporation

**Table 2:** Data collection statistics Values in parentheses correspond to the highest resolution shell.

Data collection	Lysozyme	Insulin
Number of merged data	30	13
Space group	*P*4_3_2_1_2	*R*3
Unit-cell parameters (Å)	*a* = *b* = 79.67, *c* = 37.90	*a* = *b* = 83.08, *c* = 34.39
Resolution (Å)	35.63–1.60 (1.64–1.60)	41.54–2.33 (2.39–2.33)
No. of observed reflections	116929 (5636)	9615 (168)
No. of unique reflections	16516 (1196)	2464 (102)
Completeness (%)	99.3 (99.3)	65.1 (37.0)
Multiplicity	7.1 (4.7)	3.9 (1.6)
*R*_merge_	0.064 (0.891)	0.295 (0.554)
*CC*_1/2_	0.997 (0.658)	0.890 (0.560)
