# Supplementary material for: Implementation of wedged-serial protein crystallography at PROXIMA-1
Source: J Synchrotron Radiat. 2022 Jan 17;29(Pt 2):439–46. doi: 10.1107/S1600577521013242 (PMC8900848; doi:10.1107/S1600577521013242)
Supplement: Supplementary file 2 [file s-29-00439-sup2.pdf]

JOURNAL OF SYNCHROTRON RADIATION

Volume 29 (2022)

Supporting information for article:

Implementation of wedged-serial protein crystallography at PROXIMA-1

Igor Chaussavoine, Tatiana Isabet, Robin Lener, Pierre Montaville, Ramakrishna Vasireddi and Leonard M. G. Chavas

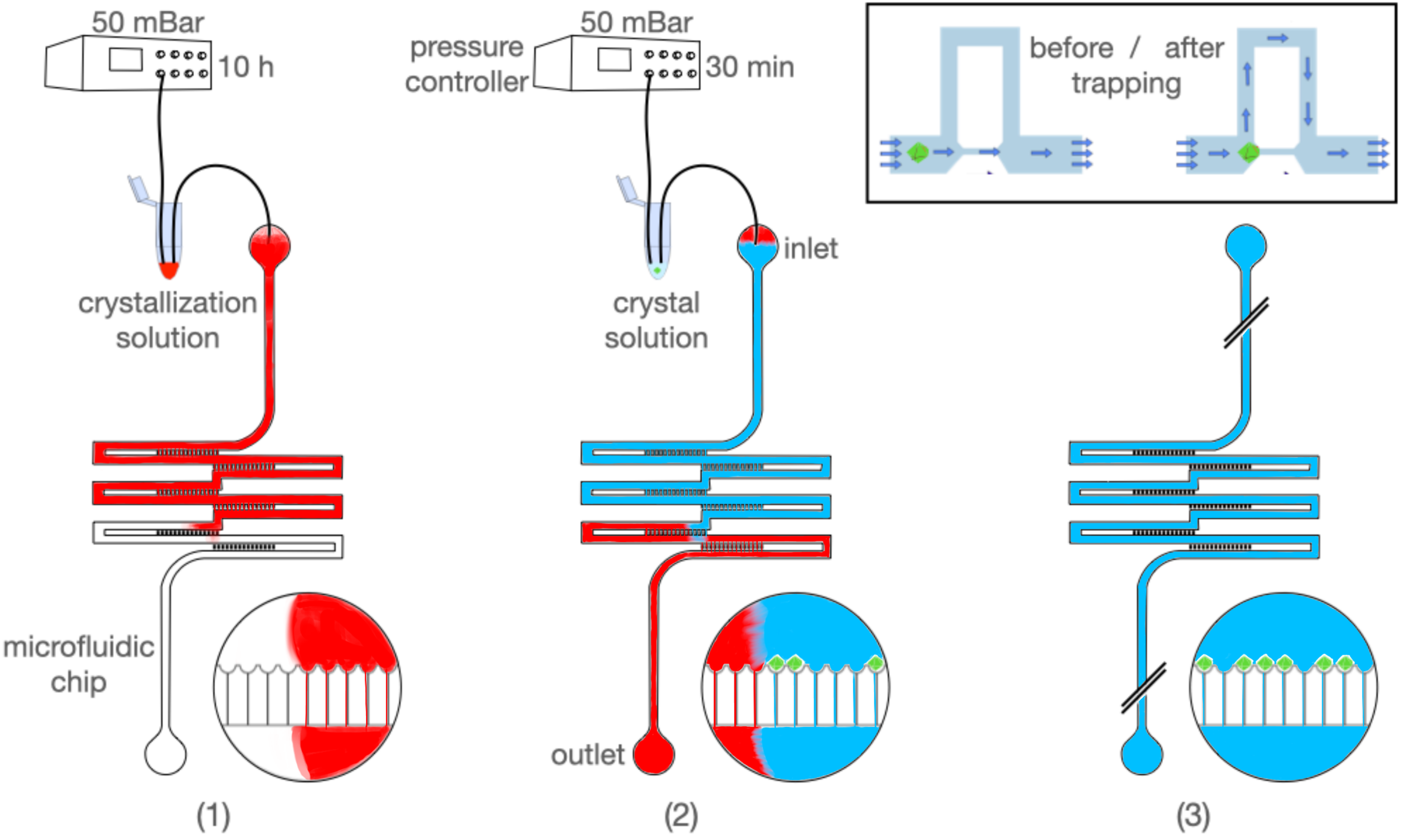

Figure S1. Schematic explanation of the sample loading process. The crystallization solution is first loaded for 10 h (pressure of 50 mBar) to assure that the microfluidic chip is fully wet and without air bubbles *(1)*. Crystals in solution are then injected for 30 min or for the full volume of sample stock *(2)* and the chip is sealed *(3)*. ***Inlet:*** crystal trapping principle, illustrating the liquid flow favored path based on the resistivity of the channels.

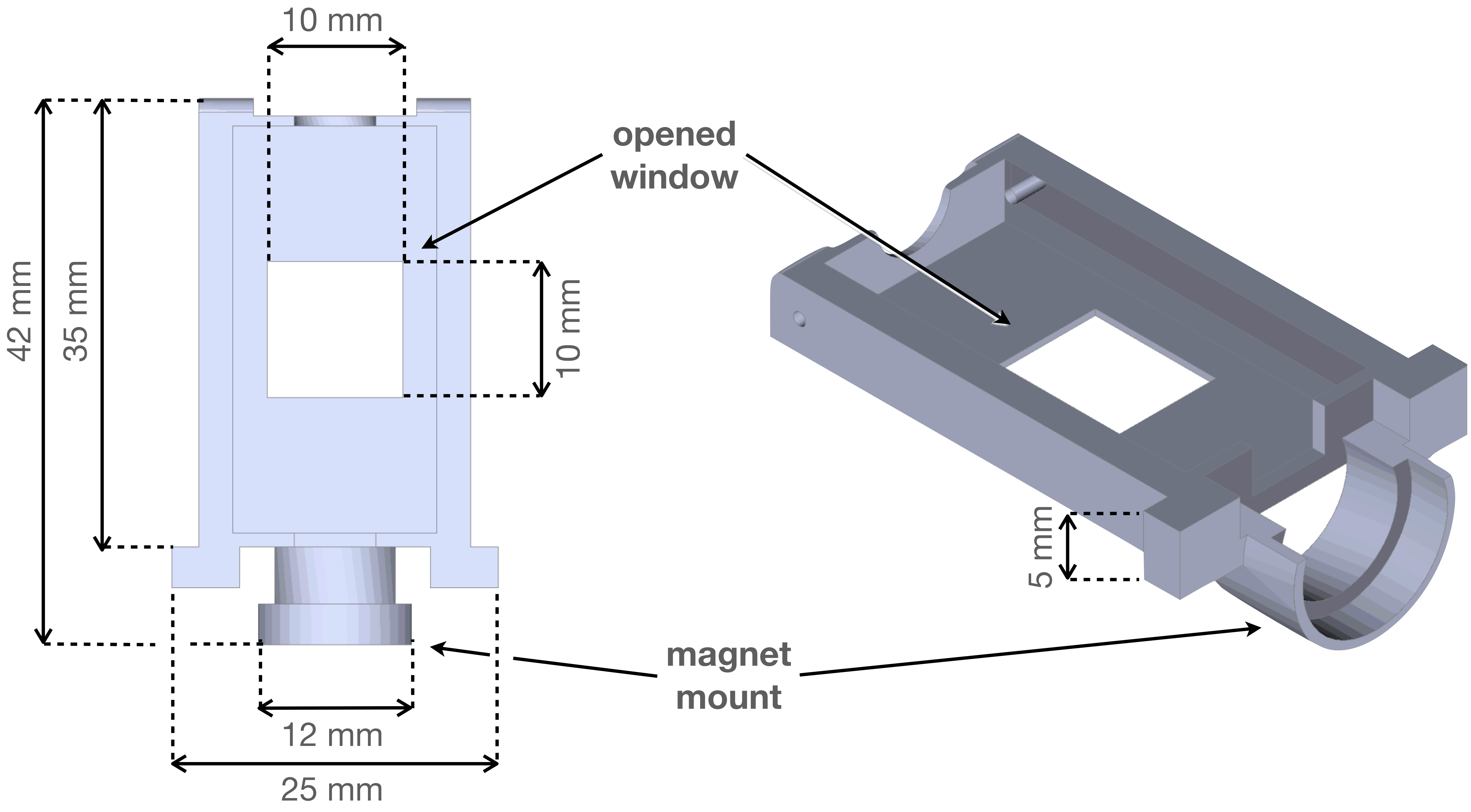

Figure S2. CAD drawing of the 3D-printed chip handling frame. The locations for fixing the magnet mount and the microfluidic chip are indicated.

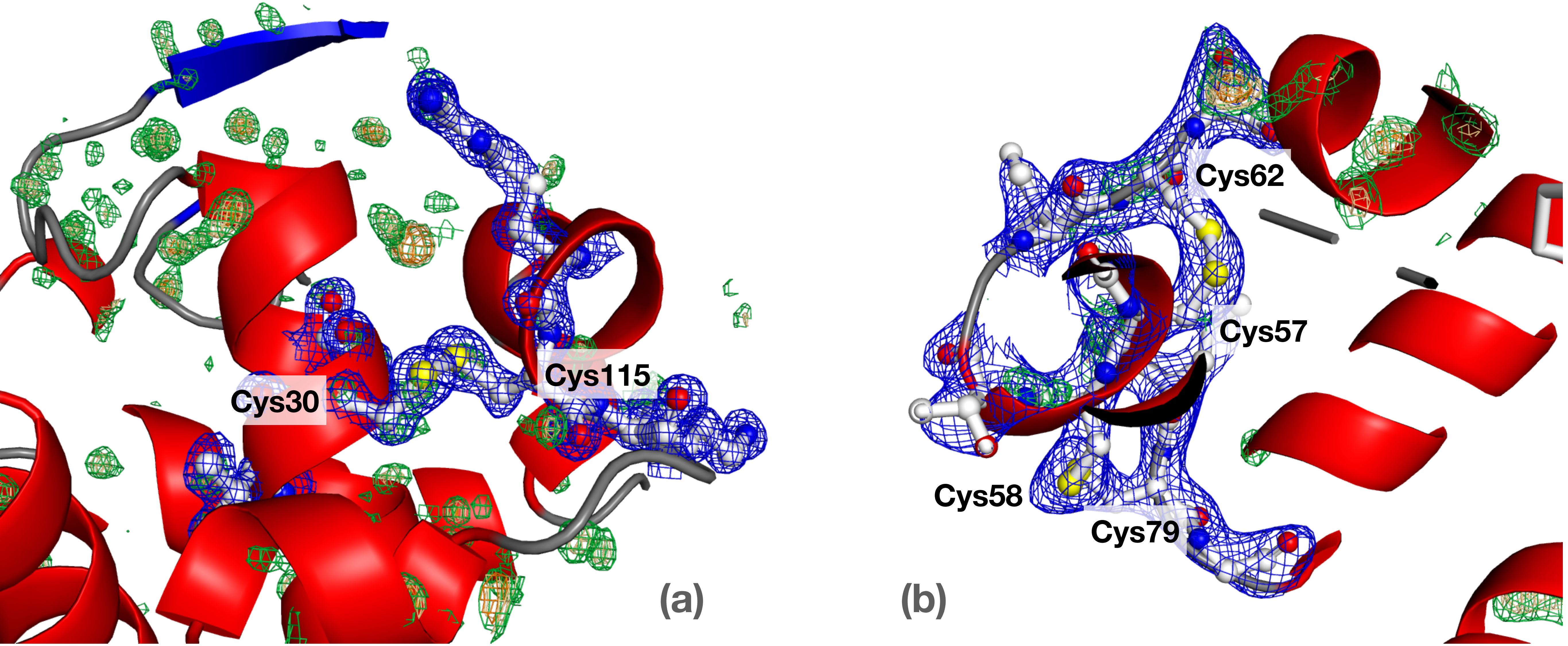

Figure S3. Electron density maps around disulfide bridges. **(a)** Lysozyme structure zoomed around the disulfide bridge Cys30-Cys115. **(b)** Insulin structure zoomed around the double disulfide bridge Cys57-Cys62 and Cys58-Cys79, respectively. The electron density maps are colored in blue for 2*Fo*-*Fc* contoured at 1.2 σ, and green, yellow and orange for *Fo*-*Fc* contoured at 2.0, 2.5 and 3.0 σ, respectively.
